# Characterizing the role of Zn cluster family transcription factor ZcfA in governing development in two *Aspergillus* species

**DOI:** 10.1371/journal.pone.0228643

**Published:** 2020-02-04

**Authors:** Ye-Eun Son, He-Jin Cho, Mi-Kyung Lee, Hee-Soo Park

**Affiliations:** 1 School of Food Science and Biotechnology, Kyungpook National University, Daegu, Republic of Korea; 2 Biological Resource Center (BRC), Korea Research Institute of Bioscience and Biotechnology (KRIBB), Jeongeup-si, Republic of Korea; 3 Department of Integrative Biology, Kyungpook National University, Daegu, Republic of Korea; Woosuk University, REPUBLIC OF KOREA

## Abstract

Filamentous fungi reproduce asexually or sexually, and the processes of asexual and sexual development are tightly regulated by a variety of transcription factors. In this study, we characterized a Zn_2_Cys_6_ transcription factor in two *Aspergillus* species, *A*. *nidulans* (*AN5859*) and *A*. *flavus* (*AFLA_046870*). *AN5859* encodes a Zn_2_Cys_6_ transcription factor, called ZcfA. In *A*. *nidulans*, Δ*zcfA* mutants exhibit decreased fungal growth, a reduction in cleistothecia production, and increased asexual reproduction. Overexpression of *zcfA* results in increased conidial production, suggesting that ZcfA is required for proper asexual and sexual development in *A*. *nidulans*. In conidia, deletion of *zcfA* causes decreased trehalose levels and decreased spore viability but increased thermal sensitivity. In *A*. *flavus*, the deletion of the *zcfA* homolog *AFLA_046870* causes increased conidial production but decreased sclerotia production; these effects are similar to those of *zcfA* deletion in *A*. *nidulans* development. Overall, these results demonstrate that ZcfA is essential for maintaining a balance between asexual and sexual development and that some roles of ZcfA are conserved in *Aspergillus* spp.

## Introduction

The genus *Aspergillus* consists of approximately 350 accepted species that are closely related [1–3]. Several species are useful for fermented food, enzyme production, and pharmaceutical purposes [4]. However, some *Aspergillus* species have detrimental effects on humans or plants [5]. Several fungi can also produce harmful secondary metabolites called mycotoxins [6, 7]. *Aspergillus flavus* is one of the key *Aspergillus* species and an agent of invasive aspergillosis in immunocompromised patients [8, 9]. *A*. *flavus* produces harmful secondary metabolites, called aflatoxins, that are potent carcinogens [10, 11]. The contamination of crops such as maize and peanuts with *A*. *flavus* causes significant economic loss [12]. Therefore, the control of *A*. *flavus* growth and aflatoxin production is crucial to the agricultural industry. To use *Aspergillus* species for the benefit of humanity, we must understand its biology. *A*. *nidulans* is a model organism for studies in fungal developmental biology and gene regulation; therefore, it is one of the best characterized *Aspergillus* species [13, 14].

The reproductive modes of *A*. *nidulans* can be divided into two types, sexual and asexual [15]. After the germ tube is formed from spores, it grows into hyphae, forming the mycelium, a web-like mass of fungal hyphae [16, 17]. After acquiring developmental competence, fungi can produce asexual or sexual developmental structures depending on environmental conditions [18, 19]. During the formation of conidiophores (asexual structures) or sexual fruiting bodies, a variety of genes and proteins participate in these processes [20, 21].

Transcription factors (TFs) are DNA-binding proteins that regulate transcription [22]. These proteins recognize specific DNA sequences in the vicinity of genes and induce or repress mRNA transcription. In the fungal genome database, 80 transcription factor families are found, and various TFs coordinate gene expression during growth and developmental processes [15, 23]. In asexual developmental processes, BrlA is a key TF for the initiation of conidiation [24, 25]. BrlA contains a C_2_H_2_ zinc finger DNA-binding domain that recognizes *brlA* response elements (BREs) in the promoter regions of certain genes, including *abaA* [26]. AbaA, a TEF1 family TF, activates mRNA expression of *wetA* and other genes in the middle phase of conidiogenesis [27, 28]. In asexual spores, a spore-specific TF WetA coordinates the mRNA expression of genes associated with spore maturation [29]. TFs BrlA, AbaA, and WetA play key roles in asexual development and are considered the core regulators of conidiation [30]. Together with these three TFs, other TFs play a vital role in the initiation of conidiation. For instance, upstream developmental activators, such as FlbB, FlbC, FlbD, and FlbE, induce *brlA* expression [31], whereas three key TFs, NsdD, SfgA, and VosA, repress conidiation [32]. In sexual developmental processes, NsdC and NsdD are key TFs that positively regulate sexual developmental processes [33–35]. With these genes, a variety of TFs are reported to be involved in sexual reproduction [21].

The Zn cluster family (Zcf) is a fungal-specific family of TFs and is the largest family of TFs known in eukaryotes [23]. Zcf TFs contain several DNA binding motifs, such as the C_2_H_2_ zinc finger, the C_4_ zinc finger, and the C_6_ zinc finger, and are involved in a variety of cellular processes in fungi [36]. For example, CrzA is a C_2_H_2_ zinc finger TF that governs calcium homeostasis, fungal growth, and detoxification in *A*. *nidulans* [37, 38]. AflR contains a Cys_6_Zn_2_ binuclear cluster motif that activates the sterigmatocystin biosynthesis gene cluster in *A*. *nidulans* [39, 40]. Although several Zcf TFs have been characterized, the functions of many other Zcf TFs have not been elucidated. Previous studies have found putative target genes of VosA, a key transcription factor for conidial maturation, in *A*. *nidulans* conidia [41]. We screened mRNA levels of putative VosA target genes in conidia and found that one gene (*AN5859*) affected the mRNA expression of these target genes when *vosA* or *velB* were deleted in conidia **(S1 Fig)**. In this study, we characterized the *AN5859* gene, which encodes the Zcf protein ZcfA in two *Aspergillus* species, *A*. *nidulans*, and *A*. *flavus*.

## Materials and methods

### Strains, media, and culture conditions

The fungal strains used in this study are listed in **Table 1**. *A*. *nidulans* strains were grown on liquid or solid minimal media with 1% glucose (MMG) for general purposes or sexual medium (SM) for sexual development [42, 43]. To confirm the effects of overexpression of the *A*. *nidulans zcfA* (*AnizcfA*) mRNA under the *alcA* promoter [44], tested strains were inoculated on MMG, MM with 100 mM threonine as the sole carbon source (MMT), or YLC (0.1% yeast extract, 1.5% lactose, 30 mM cyclopentanone) at 37°C for 5 days [45, 46]. *A*. *flavus* strains were grown on MMG with 0.1% yeast extract (MMGY) for general tests. *Escherichia coli* DH5α cells were grown in Luria-Bertani medium with ampicillin (100 μg/mL) for plasmid amplification.

**Table 1:** *Aspergillus* strains used in this study.

Strain name	Relevant genotype	References^a^
FGSC4	*A*. *nidulans* wild type	FGSC^b^
RJMP1.59	*pyrG89*; *pyroA4*	[48]
TNJ36	*pyrG89*; *AfupyrG* ^+^; *pyroA4*	[49]
THS30.1	*pyrG89*; *AfupyrG*^+^	[41]
TYE11.4~6	*pyrG89*; *pyroA4*; *ΔAnizcfA*::*AfupyrG*^+^	This study
TYE12.1	*pyrG89*; *pyroA*::*AnizcfA(p)*::*zcfA*::*FLAG3x*::*pyroA*^*c*^; *ΔAnizcfA*::*AfupyrG*^*+*^	This study
TYE17.1	*pyrG89*; *AfupyrG*^*+*^; *pyroA*::*alc(p)*::*AnizcfA*::*FLAG*::*pyroA*^*b*^;	This study
NRRL 3357	*A*. *flavus* wild type	ATCC collection
NRRL3357.5	*pyrG*^*-*^	[50]
TTJ6.1	*pyrG*^*-*^; *AfupyrG*^*+*^	This study
THJ1.1~3	*pyrG*^*-*^; *ΔAflzcfA*::*AfupyrG*^*+*^	This study

^a^ All *A*. *nidulans* strains carry the *veA*^+^ allele.

^b^ Fungal Genetic Stock Center

^c^ The 3/4 *pyroA* marker causes targeted integration at the *pyroA* locus.

For mRNA isolation, samples were collected as previously described [47]. For conidia, WT and mutant conidia were inoculated onto solid MMG and incubated for two days at 37°C. Conidia were then collected using Miracloth and stored at −80°C. For mycelia samples, WT and mutant conidia were inoculated into liquid MMG and incubated at 37°C for the indicated times. Cultured mycelia were collected, squeezed to remove moisture, and stored at -80°C. For developmental samples, submerged culture mycelia were filtered, washed, and spread in a monolayer on solid MMG, and the plates were incubated under light condition for asexual developmental induction or cultured under dark condition for sexual developmental induction.

### Construction of the *AnizcfA* mutant strains

The oligonucleotide primers used in this study are listed in **Table 2**. To generate deletion mutant strains, the double-joint PCR (DJ-PCR) method was used [51]. The 5’- and 3’-flanking regions for *AnizcfA* were amplified with primer pairs OHS220:OHS222 and OHS221:OHS223, respectively, using *A*. *nidulans* FGSC4 (wild type, WT) genomic DNA as a template. The *A*. *fumigatus pyrG* (*AfupyrG*) marker was amplified with the primer pair OHS089:OHS090 using *A*. *fumigatus* AF293 genomic DNA as a template. The final PCR construct for the *AnizcfA* deletion cassette was amplified with the OHS224:OHS225 primer pair using the three DNA fragments from the first round of PCR (the 5’- and 3’-flanking regions and the *AfupyrG* marker) as the template. The *AnizcfA* deletion cassette was introduced into *A*. *nidulans* RJMP1.59 protoplasts generated by the Vinoflow FCE lysing enzyme (Novozymes) [52, 53]. Success was confirmed by PCR followed by restriction enzyme digestion **(S2 Fig)**.

**Table 2:** Oligonucleotides used in this study.

Name	Sequence (5′ → 3′)^a^	Purpose
**OHS0220**	CCAATGAGTAGCAGCAACCTTG	5′ *AnizcfA* DF
**OHS0221**	GCGTCCGCTATCAGTTCTACCC	3′ *AnizcfA* DR
**OHS0222**	*GGCTTTGGCCTGTATCATGACTTCA* AACGTTCAAGTTGGTGAGAAGG	3′ *AnizcfA* with *AfupyrG* tail
**OHS0223**	*TTTGGTGACGACAATACCTCCCGAC* CGGCACCATATGTCCAGGAC	5′ *AnizcfA* with *AfupyrG* tail
**OHS0224**	GAGAGCTTGGAGAACAGCGAC	5′ *AnizcfA* NF
**OHS0225**	GCCATCGGACGTAGGACAAAC	3′ *AnizcfA* NR
**OHS0395**	aatt **GCGGCCGC** CCTGCAGAGTCCTACTTCCTCC	5′ *AnizcfA* with promoter & *Not*I
**OHS0396**	aatt **GCGGCCGC** CGCAGATGTAGGCGACAG	3′ *AnizcfA* with *Not*I
**OHS0741**	aatt **GGATCC** ATGGCTCCATCTTCTCGTCCT	5′ *AnizcfA* with *Bam*HI
**OHS0742**	aatt **GGATCC** TCC TGC AAA CGG CGC AGA	3′ *AnizcfA* with *Bam*HI
**OHS0475**	GTAGATCGCTTAGGTGAGCG	5′ *AflzcfA* DF
**OHS0476**	*GGCTTTGGCCTGTATCATGACTTCA* CCAACTCCACACGATCAGAC	3′ *AflzcfA* with *AfupyrG* tail
**OHS0477**	*TTTGGTGACGACAATACCTCCCGAC* CCACTCGCTCAGATGTAGGC	5′ *AflzcfA* with *AfupyrG* tail
**OHS0478**	CGGGTTGATCACCTTCATGCC	3′ *AflzcfA* DR
**OHS0479**	GTCCCACGATCATCTACCCAC	5′ *AflzcfA* NF
**OHS0480**	CATGGCTGTGCAGATCGAATAC	3′ *AflzcfA* NR
**OHS0089**	GCTGAAGTCATGATACAGGCCAAA	5′ *AfupyrG* marker_F
**OHS0090**	ATCGTCGGGAGGTATTGTCGTCAC	3′ *AfupyrG* marker_R
**OHS0044**	GTAAGGATCTGTACGGCAAC	5′ *Ani_Actin* RT_F
**OHS0045**	AGATCCACATCTGTTGGAAG	3′ *Ani_Actin* RT_R
**OHS0580**	CAAGGCATGCATCAGTACCC	5′ *Ani_brlA* RT_F
**OHS0581**	AGACATCGAACTCGGGACTC	3′ *Ani_brlA* RT_R
**OHS0777**	GGGAGCGAACAGTCTCACTA	5′ *Ani_mutA* RT_F
**OHS0778**	GTCGATTCCCGTTTCCTTGG	3′ *Ani_mutA* RT_R

^**a**^ Tail sequences are shown in italics. Restriction enzyme sites are in bold.

To generate the *ΔAnizcfA* complemented strain, the WT *AnizcfA* gene region, including its predicted promoter, was amplified with the primer pair OHS395:OHS396, digested with *Not*I, and cloned into pHS13 [54]. The resulting plasmid pYE1.1 was then introduced into the recipient *ΔAnizcfA* strain TYE11.4 to give rise to TYE12.1. Complemented strains were selected from among the transformants and screened by PCR and quantitative RT-PCR (qRT-PCR) after induction of the promoter **(S3 Fig)**.

### Construction of the *AnizcfA* overexpression strain

To generate the *alcA*(p)::*AnizcfA* fusion construct, the *AnizcfA* open reading frame (ORF) derived from genomic DNA was amplified using the primer pair OHS741:OHS742. The PCR product was then digested with *Bam*HI and cloned into pHS3, which contains the *A*. *nidulans alcA* promoter and the *trpC* terminator [54]. The resulting plasmid pYE2.1 was then introduced into TNJ36 [49]. Strains that overexpress *AnizcfA* were selected from among the transformants and screened by qRT-PCR after induction of the promoter **(S4 Fig)**.

### Construction of the *AflzcfA* deletion mutant strain

To produce the *AflzcfA* deletion cassette, the 5’ and 3’ flanking regions of the *AflzcfA* gene were amplified using the primer pairs OHS0475:OHS0476 and OHS0477:OHS0478, respectively, using *A*. *flavus* NRRL3357 genomic DNA as a template. The *AfupyrG* gene was used as a selective marker. The *AflzcfA* deletion cassette was amplified with primer pair OHS479:OHS480 and introduced into the recipient strain NRRL3357.5 [50]. Multiple (at least three) mutants were isolated and confirmed by PCR followed by restriction enzyme digestion **(S5 Fig)**.

### Nucleic acid isolation and qRT-PCR analysis

To isolate genomic DNA, approximately 10^6^ conidia of WT and mutant strains were inoculated in 2 ml liquid MMG + 0.5% yeast extract medium and allowed to grow in stationary culture at 37˚C for 24 h. The mycelial mat was collected, squeeze-dried, and genomic DNA was isolated as described [53].

For qRT-PCR analyses, total RNA isolation was carried out as previously described [55, 56]. Briefly, fresh conidia were collected and homogenized using a Mini-Bead Beater (BioSpec Products, USA) in the presence of 1 ml of TRIzol reagent (Invitrogen, USA) and 0.3 ml of zirconia/silica beads (RPI Corp., USA). The supernatant was mixed with an equal amount of cold isopropanol. After centrifugation, the supernatant was removed, and the pellet washed with 70% DEPC (diethyl pyrocarbonate)-ethanol. cDNA was synthesized from total RNA using reverse transcriptase (Promega, USA). The qRT-PCR procedure was performed using iTaq Universal SYBR Green Supermix (Bio-Rad, USA) and CFX96 Touch Real-Time PCR (Bio-Rad, USA). For an endogenous control, β-actin gene was used.

### Conidial viability assay

To test conidial viability, conidia from two-day-old and ten-day-old cultures of WT and mutant strains were collected using ddH_2_O with 0.01% Triton X-100 (Sigma, USA) [47]. After counting the number of conidia with a hematocytometer, approximately 100 conidia were inoculated onto solid MMG and incubated at 37˚C for 48 h in triplicate. After incubation, colony-forming units were counted.

### Conidia trehalose analysis

The conidia trehalose assay was performed as previously described [57]. Briefly, conidia (2 × 10^8^) from two-day-old cultures of WT and mutant strains were collected, washed with ddH_2_O, resuspended in 200 μl of ddH_2_O, and incubated at 95°C for 20 min. The supernatant was collected after centrifugation and was transferred to a new tube. An equal volume of 0.2 M sodium citrate (pH 5.5) was added, and the sample was incubated at 37°C for 8 h with or without (as a negative control) 3 mU of trehalase (Sigma, USA). The amount of glucose generated from the trehalose was assayed with a Glucose Assay Kit (Sigma, USA) in triplicate.

### Thermal stress response assay

Thermal tolerance tests were carried out as previously described [57, 58]. Briefly, approximately 10^3^ conidia per ml were incubated for 15 min at 55°C. The conidial suspension was then diluted with ddH_2_O, and the conidia were inoculated onto solid MMG. After incubation at 37°C for 48 h, colony numbers were counted and calculated as a survival ratio relative to counts obtained from the untreated control.

### Sterigmatocystin (ST) extraction and thin-layer chromatography (TLC) analysis

Briefly, 10^5^ conidia of each strain were inoculated into 5 ml liquid complete medium (CM) and cultured at 30°C for 7 days. Secondary metabolites were extracted by adding 5 ml of CHCl_3_, and the organic phase was separated by centrifugation and transferred to new glass vials. The organic phase was evaporated in an oven, and the residue was resuspended in 100 μl of CHCl_3_. Secondary metabolites were spotted onto a TLC silica plate that included a fluorescence indicator (Kieselgel 60, 0.25 mm; Merck) and resolved in chloroform:ethyl acetate (9:1, v/v). The images of TLC plates were captured following exposure to ultraviolet illumination at 366 nm. The TLC plate was then treated with 1% aluminum hydroxide hydrate (Sigma, USA). Quantification of ST spot intensity (366 nm on the TLC plates) was calculated using ImageJ software. Experiments were performed in triplicate per strain.

### Microscopy

Photographs of colonies were taken with a Pentax MX-1 digital camera. Photomicrographs were taken with a Zeiss Lab.A1 microscope equipped with an AxioCam 105c camera and AxioVision (Rel. 4.9) digital imaging software.

### Statistical analysis

Statistical differences between WT and mutant strains were evaluated by Student’s unpaired *t*-test. Mean ± standard deviation (SD) are shown. P values < 0.05 were considered to be significant. (*, *p* < 0.05; **, *p* < 0.01; ***, *p* < 0.001).

## Results

### ZcfA homologs in *Aspergillus* species

**Fig 1:**
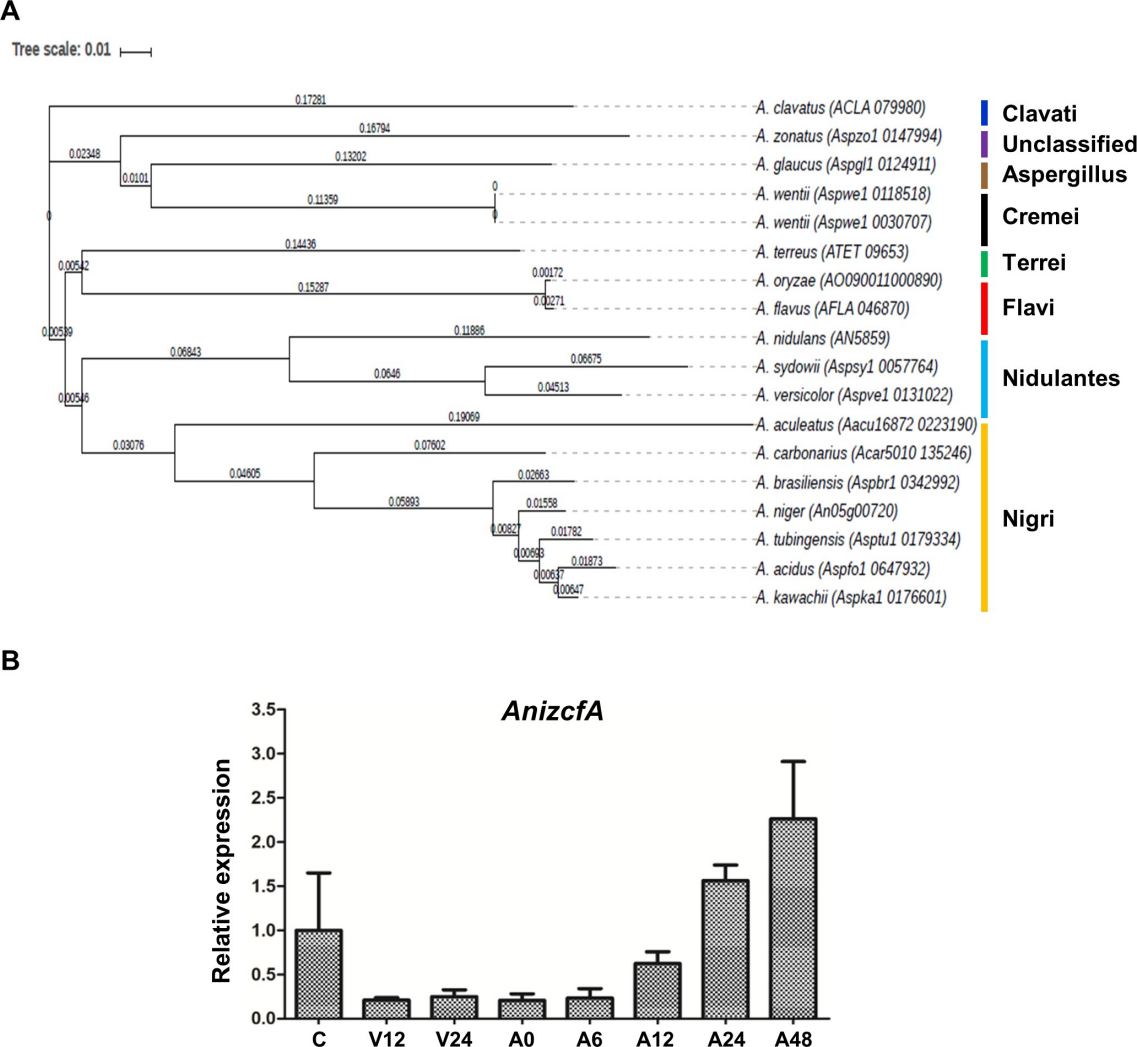
mRNA expression and homology analysis of *zcfA*. **(A)** A phylogenetic tree of ZcfA homolog proteins identified in *Aspergillus* species including *A*. *oryzae* (AO090011000890), *A*. *flavus* (AFL2T 05600), *A*. *aculeatus* (Aacu16872 022319), *A*. *carbonarius* (Acar5010 135246), *A*. *brasiliensis* (Aspbr1 0342992), *A*. *niger* (An05g00720), *A*. *tubingensis* (Asptu1 0179334), *A*. *acidus* (Aspfo1 0647932), *A*. *kawachii* (Aspka1 0176601), *A*. *terreus* (ATET 09653), *A*. *nidulans* (AN5859), *A*. *sydowii* (Aspsy1 0057764), *A*. *versicolor* (Aspve1 0131022), *A*. *clavatus* (ACLA 079980), *A*. *zonatus* (Aspzo1 0147994), *A*. *glaucus* (Aspgl1 0124911), and *A*. *wentii* (Aspwe1 0118518; Aspwe1 0030707). A phylogenetic tree of ZcfA homologs was generated by MEGA 5 software (http://www.megasoftware.net/) using the alignment data from ClustalW2. The tree results were submitted to iTOL (http://itol.embl.de/) to generate the figure. **(B)** mRNA levels of *zcfA* during the lifecycle of *A*. *nidulans*. Time (h) of incubation in liquid culture (V) or in post asexual developmental induction (A) is shown. Conidia (asexual spores) are indicated as C.

Previous studies reported that the Zn cluster family TFs are major TFs in fungi and are involved in various biological processes [23, 36]. In this study, we characterized the Zcf TF ZcfA (*AN5859*) in the model fungus *A*. *nidulans*. *AN5859* encodes a protein that contains a GAL4-like Zn_2_Cys_6_ binuclear cluster DNA-binding domain at the N-terminus and a fungal transcription factor regulatory middle homology region (MHR). To find homologs in other *Aspergillus* species, we screened the *Aspergillus* genome database. Interestingly, although most *Aspergillus* species contain ZcfA homologs, a ZcfA homolog is not seen in *Aspergillus* strains belonging to the section Fumigati, such as *A*. *fumigatus* **(Fig 1A)**. To characterize the *zcfA* gene in *A*. *nidulans*, we first investigated the expression of *AnizcfA* mRNA during the fungal lifecycle. *AnizcfA* mRNA levels increase 12 h after induction of asexual development and decrease in conidia **(Fig 1B)**.

### The role of *AnizcfA* in fungal growth and asexual development

**Fig 2:**
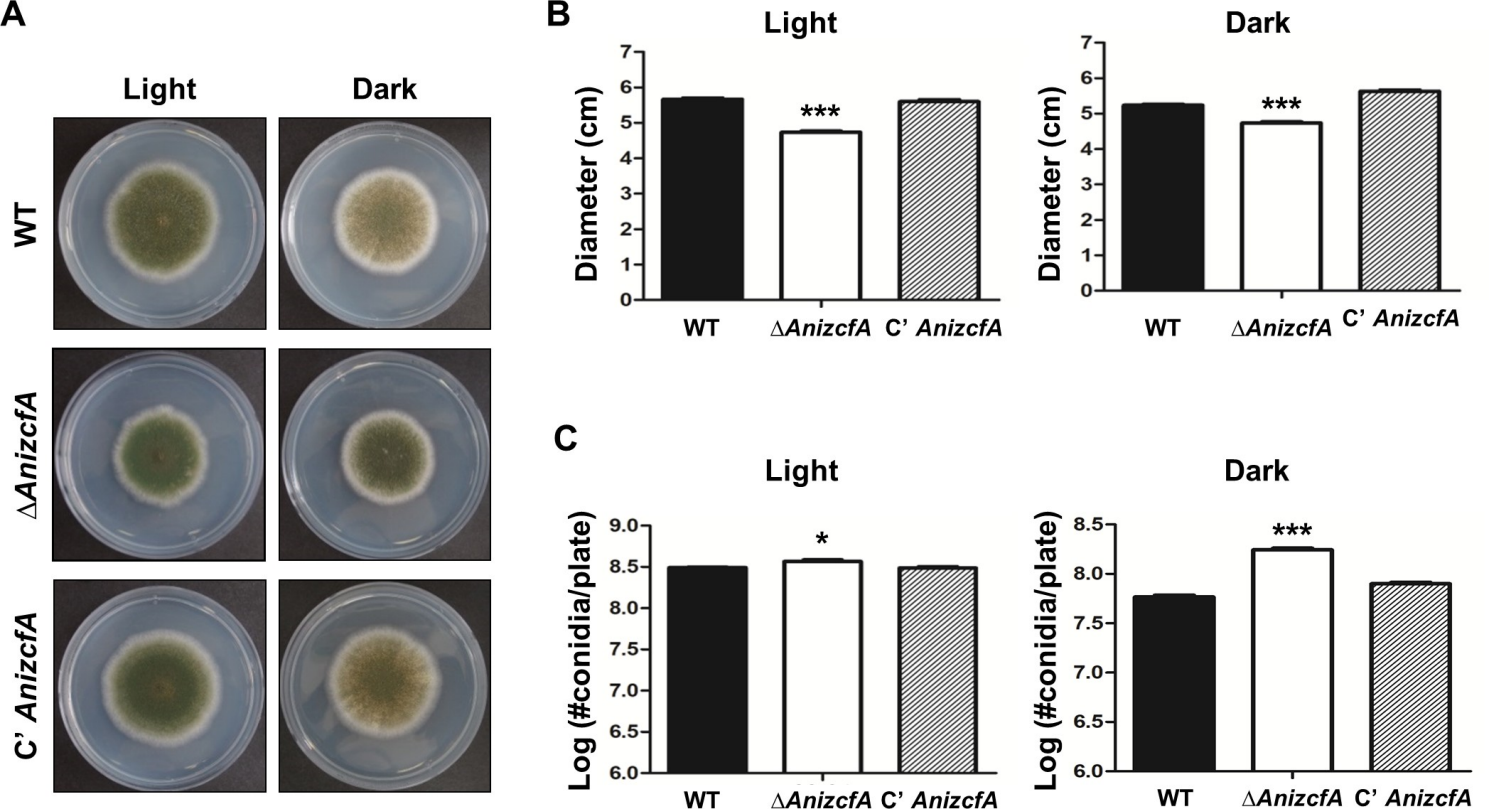
Phenotypic analysis of the Δ*AnizcfA* mutant. **(A)** Colony photographs of WT (FGSC4), Δ*AnizcfA* (TYE11.4), and C’ *AnizcfA* (TYE12.1) strains that were point-inoculated on solid MMG and grown for five days under light or dark conditions. **(B)** Quantitative analysis of colony diameter for WT (FGSC4), Δ*AnizcfA* (TYE11.4), and C’ *AnizcfA* (TYE12.1) strains shown in (A). Error bars indicate the standard error of the mean in triplicate samples (Control vs. Δ*AnizcfA* strains, *** *p* ≤ 0.001). **(C)** Quantitative analysis of asexual spore production of WT (FGSC4), Δ*AnizcfA* (TYE11.4), and the complemented (TYE12.1) strains shown in (A) (Control vs. Δ*AnizcfA* strains, * *p* ≤ 0.05; *** *p* ≤ 0.001).

To further characterize *AnizcfA*, an *AnizcfA* deletion (Δ*AnizcfA*) mutant and complemented strains were generated, and their phenotypes examined. WT, Δ*AnizcfA*, and complemented (C’ *AnizcfA*) strains were point-inoculated onto MM solid media, incubated under light and dark conditions, and then colony growth and production of asexual spores were assessed **(S6 Fig)**. As shown in **Fig 2**, the colony diameter of the Δ*AnizcfA* strain under both light and dark conditions was less than those of WT and complemented strains. However, the Δ*AnizcfA* strain produced more conidia per plate under both dark and light conditions. These results indicate that ZcfA is required for proper growth and conidiation in *A*. *nidulans*.

**Fig 3:**
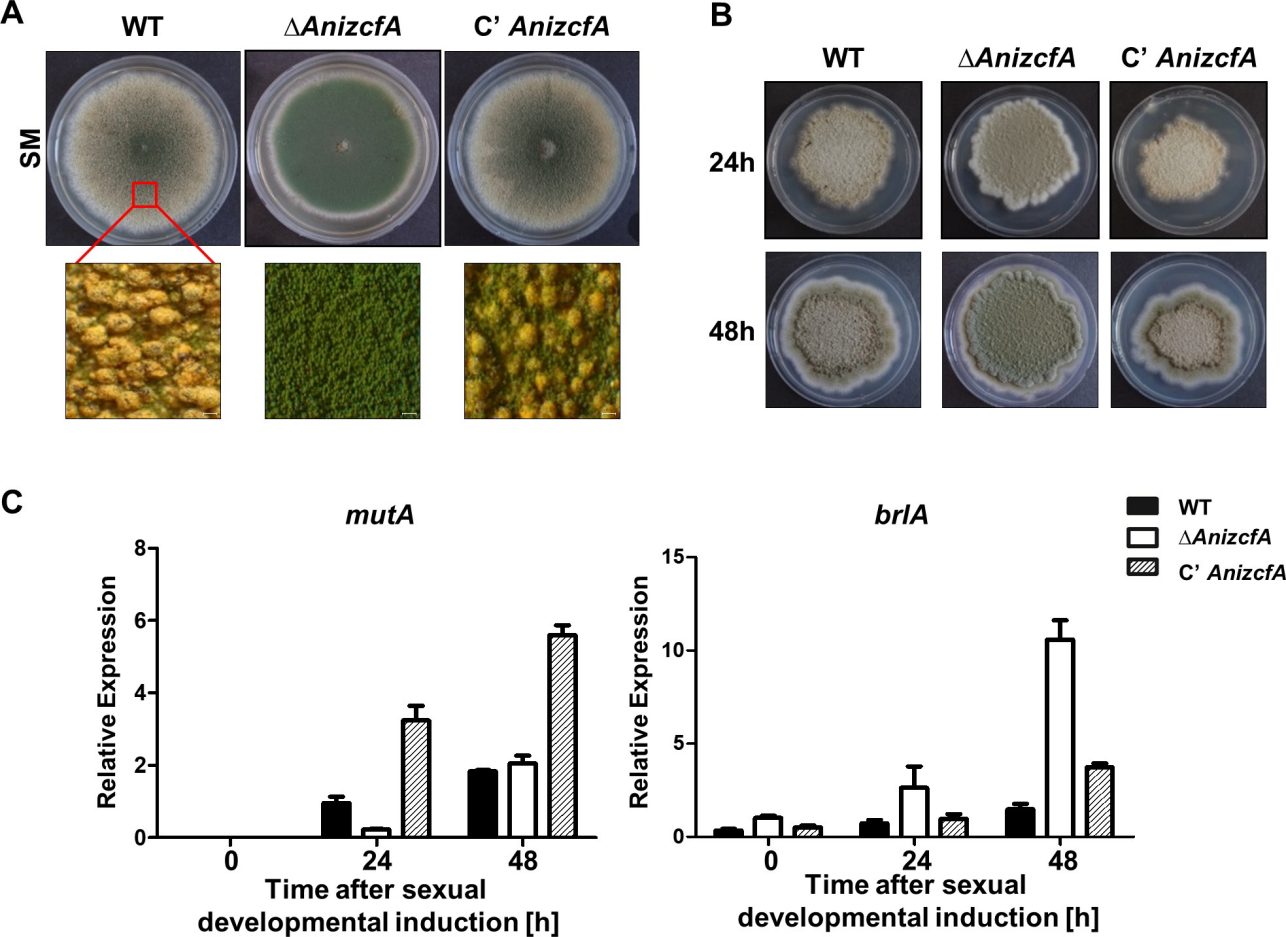
Sexual development phenotypes of the Δ*zcfA* mutant. **(A)** Colony photographs of WT (FGSC4), Δ*AnizcfA* (TYE11.4), and C’ *AnizcfA* (TYE12.1) strains that were point-inoculated on solid SM and grown for seven days under dark conditions. The bottom panel shows close-up views of the center of the plates. **(B)** Phenotypes of WT (FGSC4), Δ*AnizcfA* (TYE11.4), and C’ *AnizcfA* (TYE12.1) strains after induction of sexual development. **(C)** qRT-PCR analyses of *mutA* and *brlA* mRNA levels in WT (FGSC4), Δ*AnizcfA* (TYE11.4), and C’ *AnizcfA* (TYE12.1) strains. Numbers indicate the time (h) after the induction of sexual development.

To further test the role of ZcfA in developmental processes, the Δ*AnizcfA* strain was inoculated onto SM and incubated under dark conditions. The WT and complemented strains produced more sexual fruiting bodies under these conditions, but the Δ*AnizcfA* strain produced asexual spores predominantly and a negligible number of sexual fruiting bodies **(Fig 3A)**. To test whether the deletion of *AnizcfA* affects the expression of genes involved in asexual and sexual development, submerged culture mycelia of WT, Δ*AnizcfA*, and C’ *AnizcfA* strains were spread onto solid MMG and cultured under dark conditions to induce sexual development **(Fig 3B)**. After cultivation, the samples were collected, and the mRNA expression of α-1,3-mutanase (*mutA*), a gene that is specifically expressed in Hülle cells [59], and *brlA*, a key gene in conidial initiation [24] were measured. As shown in **Fig 3C**, deletion of *AnizcfA* leads to reduced (or delayed) mRNA expression of *mutA*. In contrast, mRNA expression of *brlA* is induced in the Δ*AnizcfA* strain. Taken together, these results suggest that ZcfA is critical for fungal growth and maintaining a balance between asexual and sexual development in *A*. *nidulans*.

### Role of *Ani*ZcfA in conidial viability and trehalose content

**Fig 4:**
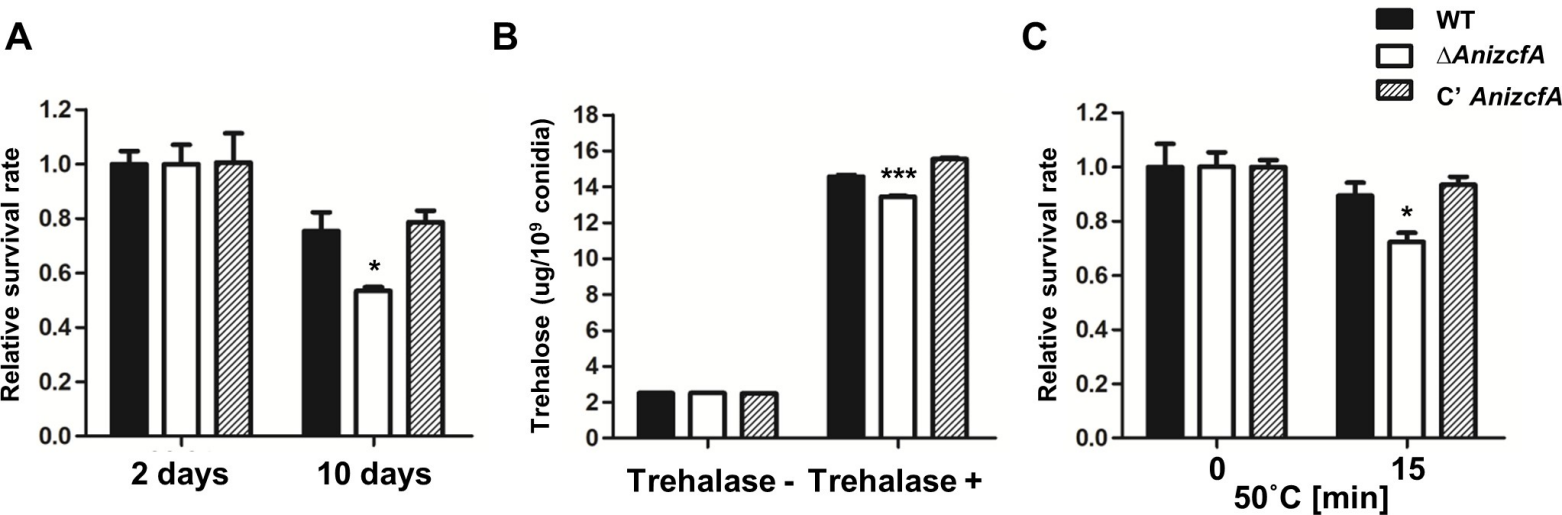
Role of *Ani*ZcfA in conidial viability and trehalose content. **(A)** Viability of the conidia of WT (FGSC4), Δ*AnizcfA* (TYE11.4), and C’ *AnizcfA* (TYE12.1) strains grown for 2 and 10 days (WT vs. Δ*AnizcfA* strains, * *p* ≤ 0.05). **(B)** Amount of trehalose per 10^8^ conidia in the two-day-old conidia of WT (FGSC4), Δ*AnizcfA* (TYE11.4), and C’ *AnizcfA* (TYE12.1) strains (measured in triplicate) (WT vs. Δ*AnizcfA* strains, *** *p* ≤ 0.001). **(C)** Tolerance of WT (FGSC4), Δ*AnizcfA* (TYE11.4), and C’ *AnizcfA* (TYE12.1) conidia to thermal stress (50°C, triplicate measurements) (WT vs. Δ*AnizcfA* strains, * *p* ≤ 0.05).

To investigate the role of *Ani*ZcfA in conidia, we first examined the viability of conidia produced by the Δ*AnizcfA* strain. As shown in **Fig 4A**, the viability of Δ*AnizcfA* conidia decreased at ten days compared with that at two days. We then measured the amount of trehalose produced by WT, Δ*AnizcfA*, and C’ strains and found that trehalose levels in Δ*AnizcfA* conidia were slightly lower than those in WT and complemented conidia (**Fig 4B)**. A previous study reported that conidial trehalose affects tolerance to thermal stresses in *A*. *nidulans* conidia [57]. To determine whether the lower level of trehalose in Δ*AnizcfA* conidia alters their stress response, thermal tolerance was assayed. The Δ*AnizcfA* conidia were more sensitive to heat stress than WT and C' conidia **(Fig 4C)**. Although the phenotype of the Δ*AnizcfA* conidia is only slightly changed, these results suggest that ZcfA plays a key role in conidial viability and trehalose levels in *A*. *nidulans*.

### Overexpression of *AnizcfA* leads to increased conidia production

**Fig 5:**
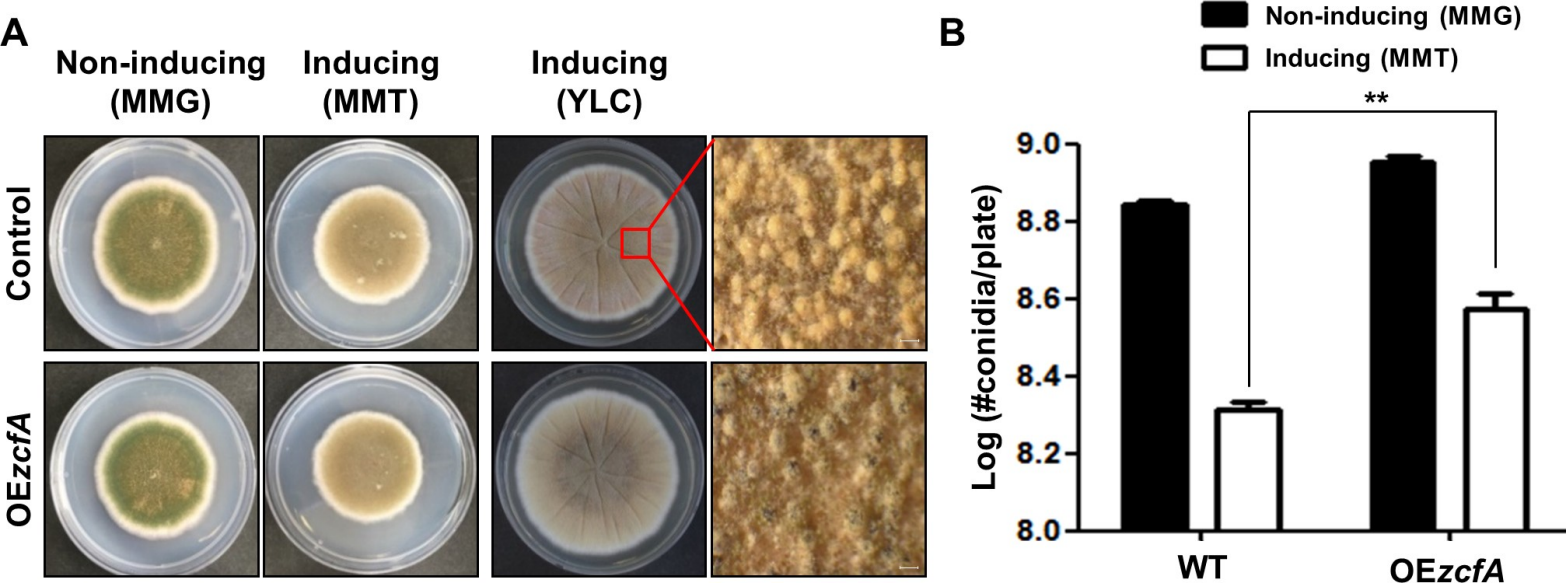
Effect of *AnizcfA* overexpression. **(A)** Control (THS30) and OE*zcfA* (*AnizcfA* overexpression, TYE17.1) strains were point-inoculated on solid MMG (non-inducing), MMT (100 mM threonine, inducing), and YLC (inducing) media and photographed at day 4 or 7. **(B)** Quantitative analysis of conidial production in control (THS30) and OE*zcfA* (TYE17.1) strains shown in (A) (** *p* ≤ 0.01).

To further investigate the role of *AnizcfA* in fungal development, a strain that overexpresses *AnizcfA* was developed. Control and *AnizcfA* overexpression mutant (OE*zcfA*) strains were inoculated under non-inducing and inducing conditions **(Fig 5)**. Under conditions that induce the *alcA* promoter (MMT medium), overexpression of *AnizcfA* results in increased production of asexual spores. In YLC media (another *alcA*-inducing condition), *AnizcfA* overexpression strains produced fewer sexual fruiting bodies than WT. These results support the idea that *Ani*ZcfA is essential for proper developmental processes.

### Deletion of *AnizcfA* alters sterigmatocystin production

**Fig 6:**
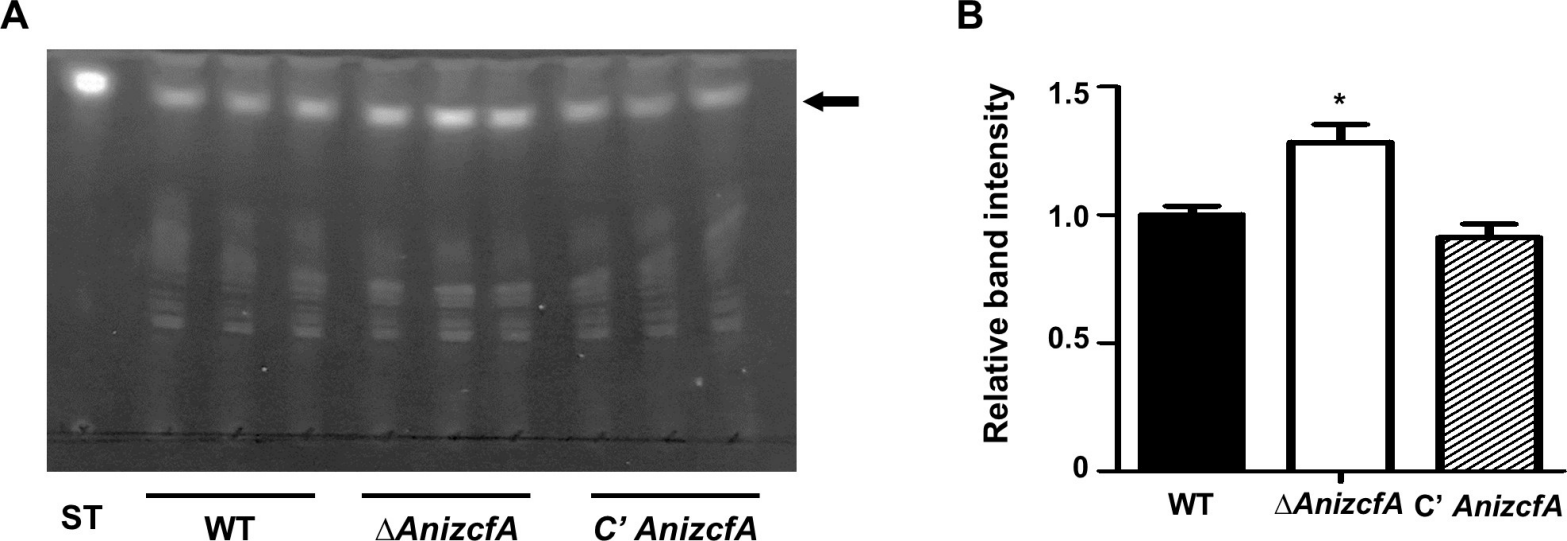
Sterigmatocystin production in the Δ*zcfA* mutant. **(A)** Thin-layer chromatography (TLC) analysis of sterigmatocystin (ST) produced by WT (FGSC4), Δ*AnizcfA* (TYE11.4), and C’ *AnizcfA* (TYE12.1) strains. **(B)** Intensity of the ST bands using ImageJ software (WT vs. Δ*AnizcfA* strains, * *p* ≤ 0.05).

Because fungal development is associated with secondary metabolism [60], we hypothesized that *zcfA* might be involved in secondary metabolism. To test whether *zcfA* affects ST production in *A*. *nidulans*, secondary metabolites were extracted from WT, Δ*AnizcfA*, and C’ strains. Three independent samples were extracted, spotted, and loaded onto TLC plates. The Δ*AnizcfA* strain produced more ST than WT or complemented strains **(Fig 6)**. However, the overexpression of *zcfA* did not affect ST production **(S7 Fig)**. This suggests that ZcfA influences the production of ST in *A*. *nidulans*.

### ZcfA homolog is required for development in *A*. *flavus*

**Fig 7:**
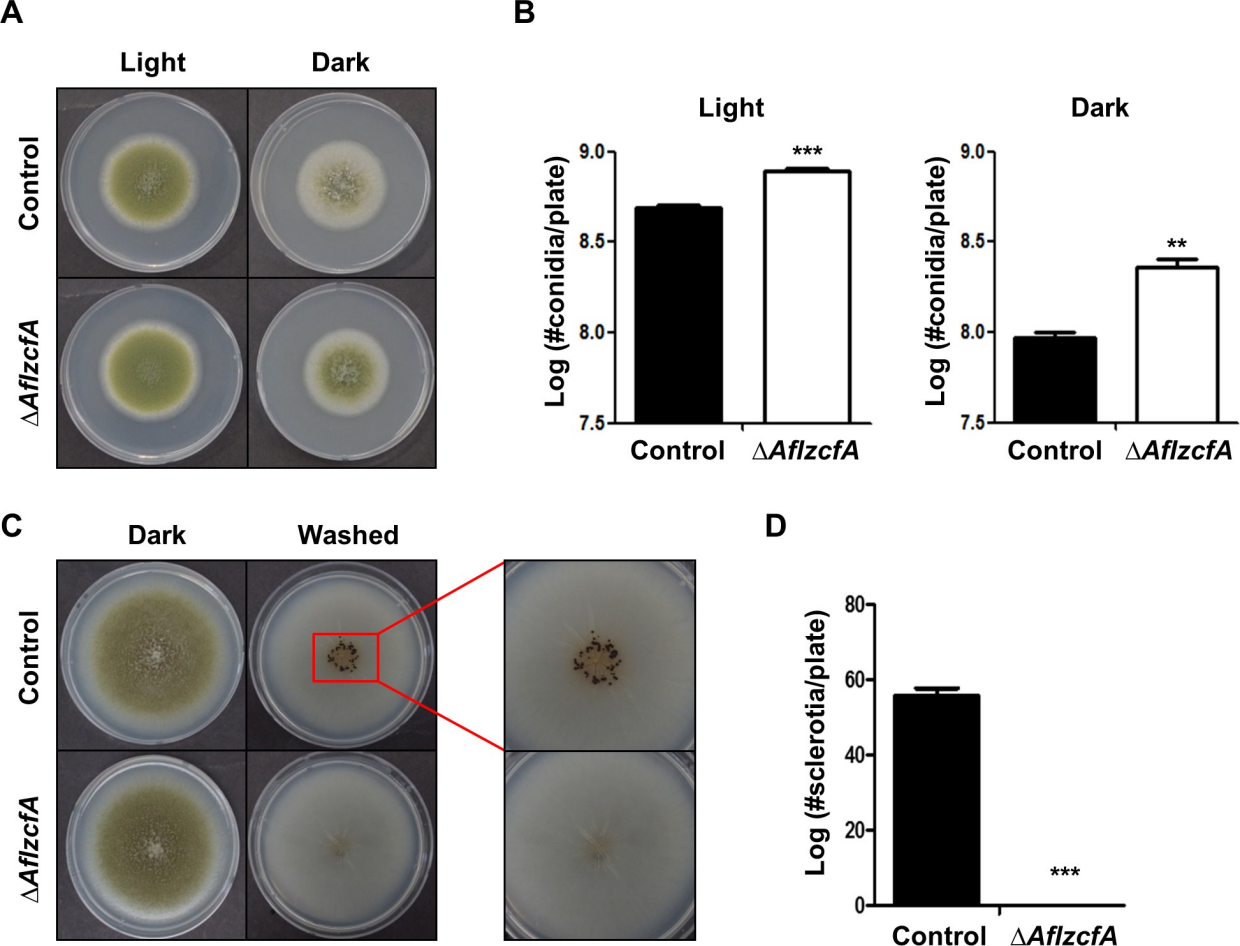
Roles of ZcfA in *A*. *flavus*. **(A)** Colony photographs of control (TTJ6.1) and Δ*AflzcfA* (THJ1.1) strains that were point-inoculated on solid MMGY and grown for five days under light or dark conditions. **(B)** Quantitative analysis of the colony diameter of the control (TTJ6.1) and Δ*AflzcfA* (THJ1.1) strains shown in (A) (Control vs. Δ*AflzcfA* strains, ** *p* ≤ 0.01; *** *p* ≤ 0.001). **(C)** Colony morphology of control (TTJ6.1) and Δ*AflzcfA* (THJ1.1) strains were point-inoculated on solid MMGY and grown at 37°C under dark conditions for 7 days. The plates were washed with 100% ethanol to enable the visualization of sclerotia. **(D)** Quantitative analysis of sclerotia production of control (TTJ6.1) and Δ*AflzcfA* (THJ1.1) strains shown in (C) (Control vs. Δ*AflzcfA* strains, *** *p* ≤ 0.001).

As mentioned above, ZcfA is conserved in most *Aspergillus* species and plays a crucial role in fungal development in the model organism, *A*. *nidulans*. Therefore, we hypothesized that ZcfA homologs might play similar roles in other *Aspergillus* species and tested this hypothesis in *A*. *flavus*. We generated an *A*. *flavus zcfA* deletion mutant strains (Δ*AflzcfA*) and examined their developmental phenotypes **(S8 Fig)**. WT and Δ*AflzcfA* mutant strains were inoculated onto MMGY, and the plates were incubated under light and dark conditions **(Fig 7A)**. The deletion of *AflzcfA* causes increased production of asexual spores in both light and dark conditions **(Fig 7B)**. In addition, the Δ*AflzcfA* mutant cannot produce sclerotia, or sexual structures, in this condition **(Fig 7C and 7D)**. These results suggest that ZcfA is essential for proper fungal development and that its function is similar among *Aspergillus* species that contain a homolog of ZcfA.

## Discussion

Zcf proteins are a family of fungal-specific TFs that are the largest known family of TFs among eukaryotes [36]. In the *A*. *nidulans* genome, approximately 50 proteins that contain C_6_ zinc finger motifs have been identified, and these Zcf proteins regulate the expression of genes associated with primary and secondary metabolism. For example, AflR acts as an activator for the ST biosynthesis gene cluster [39]. AmyR is also a Zn_2_Cys_6_ transcription activator that regulates amylolytic gene expression [61, 62]. In this study, we characterized the Zn_2_Cys_6_ transcription factor ZcfA in two *Aspergillus* species. In both species, Δ*zcfA* mutant strains exhibited increased conidial production and decreased formation of sexual fruiting bodies, suggesting that ZcfA may act as an asexual development repressor or a sexual development activator. To further examine the role of ZcfA, phenotypes, *zcfA* overexpression strains were examined in *A*. *nidulans*. Contrary to our expectation, overexpression of *zcfA* increased asexual spore production but decreased the production of sexual fruiting bodies. This result implies that ZcfA may not be an asexual development repressor or a sexual development activator. Our results suggest that ZcfA appears to be essential for proper fungal development in *A*. *nidulans*.

As shown in **Fig 1A**, *zcfA* mRNA expression in *A*. *nidulans* increases during asexual development, suggesting that *zcfA* expression might regulate other regulators of asexual development, such as BrlA or AbaA. We examined the *zcfA* promoter region and found several AbaA response elements (AREs, 5’-CATTCY-3’) [28] but no BrlA response elements (BRE) [26]. Further *zcfA* expression analysis will be conducted to elucidate how *zcfA* expression is regulated during asexual development. In conidia, mRNA expression of *zcfA* was decreased (**Fig 1**). It is possible that transcription factors (e.g., WetA, VosA, and VelB) that are important for spore maturation are involved in the expression of *zcfA* mRNA. We have measured *zcfA* transcript levels in Δ*wetA*, Δ*vosA*, and Δ*velB* mutant conidia. Our preliminary data **(S1 Fig)** and published data [29] show that *zcfA* mRNA levels in conidia from these mutants are increased compared to WT. Overall, these results indicate that *zcfA* mRNA levels may be regulated by certain asexual regulators in *A*. *nidulans*.

In conidia, ZcfA affects spore viability, trehalose contents, and thermal tolerance in *A*. *nidulans*. It appears that these roles of ZcfA are conserved in the development of two *Aspergillus* species; therefore, we hypothesize that the roles of ZcfA might be conserved in conidia. We examined the role of ZcfA in *A*. *flavus* conidia and found that trehalose contents, conidial viability, and stress response of Δ*AflzcfA* mutant conidia were similar to those of WT conidia. These results suggest that the roles of ZcfA role in conidia are not conserved among *Aspergillus* species.

In summary, we characterized the Zn cluster family transcription factor ZcfA in the model organism *A*. *nidulans* and *A*. *flavus*. In both species, ZcfA affects fungal differentiation. The deletion of *zcfA* causes a decrease (or lack of) in the formation of sexual fruiting bodies in both *Aspergillus* species. ZcfA does not act as a repressor or activator of fungal development, yet it is required for proper asexual and sexual development in *Aspergillus* species. In *A*. *nidulans*, ZcfA is involved in spore viability and secondary metabolism. Although the roles of ZcfA have been characterized in *A*. *nidulans*, the regulatory mechanisms of ZcfA function are not yet known. Further genomic and biochemical studies will provide insight into the regulatory mechanisms of ZcfA in *Aspergillus* species.

## Supporting information

S1 Fig Levels of *zcfA* mRNAs. **(A)** *AnizcfA* mRNA levels in WT, *ΔAnivosA*, and *ΔAnivelB* mutant conidia. **(B)** *AflzcfA* mRNA levels in WT, *ΔAflvosA*, and *ΔAflvelB* mutant conidia. To calculate the expression levels of the *AnizcfA* and *AflzcfA* genes, the 2^-ΔΔCT^ method was used, with β-actin as an endogenous control. Statistical differences between WT and mutant strains were analyzed by the Student’s unpaired t-test. Error bars indicate the standard error of the mean in triplicate samples.
(TIF)

S2 Fig Verification of the *ΔAnizcfA* mutant. **(A)** Diagram of the strategy used to generate the *ΔAnizcfA* mutant strain. Arrows indicate the primers used to verify the mutant strain. **(B)** PCR verification of the *ΔAnizcfA* mutant strain. **(C)** Restriction enzyme digestion verification of the *ΔAnizcfA* mutant strain.
(TIF)

S3 Fig Verification of the C’A*nizcfA* strain. **(A)** PCR verification of the C’*AnizcfA* strain. **(B)** qRT-PCR verification of the C’*AnizcfA* strain. To calculate the expression levels of *AnizcfA*, the 2^-ΔΔCT^ method was used, with β-actin as an endogenous control. Statistical differences between WT and mutant strains were analyzed by the Student’s unpaired t-test. Error bars indicate the standard error of the mean in triplicate samples.
(TIF)

S4 Fig Verification of the OE*zcfA* mutant strains. **(A)** qRT-PCR verification of the OE*zcfA* strains. **(B)** Phenotype of the OE*zcfA* mutant strains. To calculate the expression levels of *AnizcfA*, the 2^-ΔΔCT^ method was used, with β-actin as an endogenous control. Statistical differences between WT and mutant strains were analyzed by the Student’s unpaired t-test. Error bars indicates the standard error of the mean in triplicate samples.
(TIF)

S5 Fig Verification of the *ΔAflzcfA* mutant. **(A)** Diagram of the strategy for to generate the *ΔAflzcfA* mutants. Arrows indicate the primers used to verify the mutant strain. **(B)** PCR verification of the *ΔAflzcfA* mutant strain. **(C)** Restriction enzyme digestion verification of the *ΔAnizcfA* mutant strain.
(TIF)

S6 Fig Phenotype of the *ΔAnizcfA* mutant strains. Colony photographs of WT (FGSC4) and Δ*AnizcfA* (TYE11.4–6) that were point-inoculated on solid MMG and grown for five days under light or dark conditions.
(TIF)

S7 Fig Sterigmatocystin production in the OE*zcfA* mutant. **(A-B)** Thin-layer chromatography (TLC) analysis of sterigmatocystin (ST) produced by WT and OE*zcfA* strains in MMT **(A)** or YLC **(B)** inducing media.
(TIF)

S8 Fig Phenotype of the *ΔAflzcfA* mutant strains. **(A)** Colony photographs of control (TTJ6.1) and Δ*AflzcfA* (THJ1.1–3) strains that were point-inoculated on solid MMGY and grown for five days under light or dark conditions. **(B)** Colony morphology of control (TTJ6.1) and Δ*AflzcfA* (THJ1.1–3) strains were point-inoculated on solid MMGY and grown at 37°C under dark conditions for 7 days. The plates were washed with 100% ethanol to visualize sclerotia.
(TIF)
