# Supplementary figures and images for: Characterizing the role of Zn cluster family transcription factor ZcfA in governing development in two Aspergillus species
Source: PLoS One. 2020 Feb 4;15(2):e0228643. doi: 10.1371/journal.pone.0228643 (PMC6999877; doi:10.1371/journal.pone.0228643)

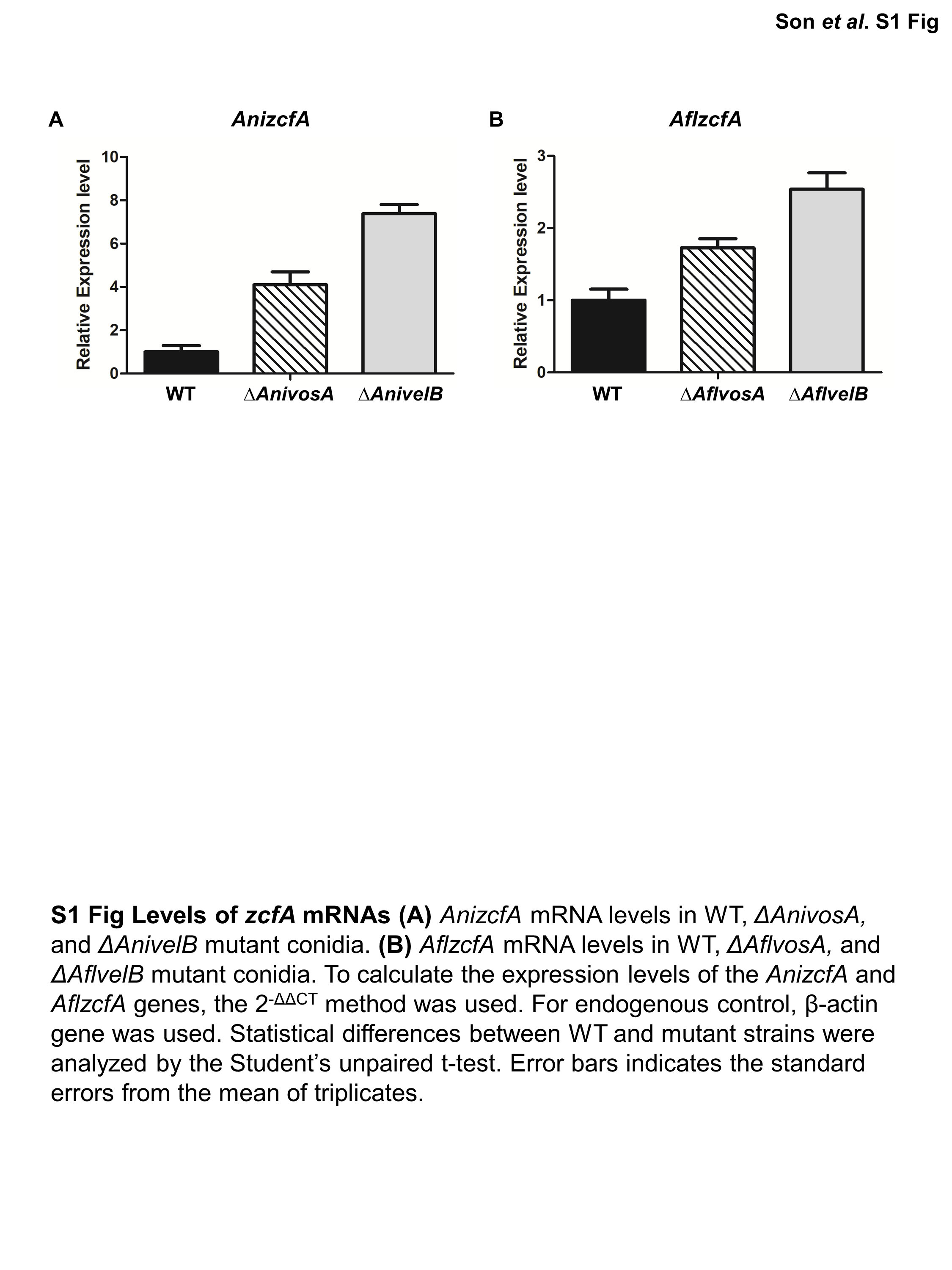

Supplement: S1 Fig — (A) AnizcfA mRNA levels in WT, ΔAnivosA, and ΔAnivelB mutant conidia. (B) AflzcfA mRNA levels in WT, ΔAflvosA, and ΔAflvelB mutant conidia. To calculate the expression levels of the AnizcfA and AflzcfA genes, the 2-ΔΔCT method was used, with β-actin as an endogenous control. Statistical differences between WT and mutant strains were analyzed by the Student’s unpaired t-test. Error bars indicate the standard error of the mean in triplicate samples. (TIF) [file pone.0228643.s001.TIF]

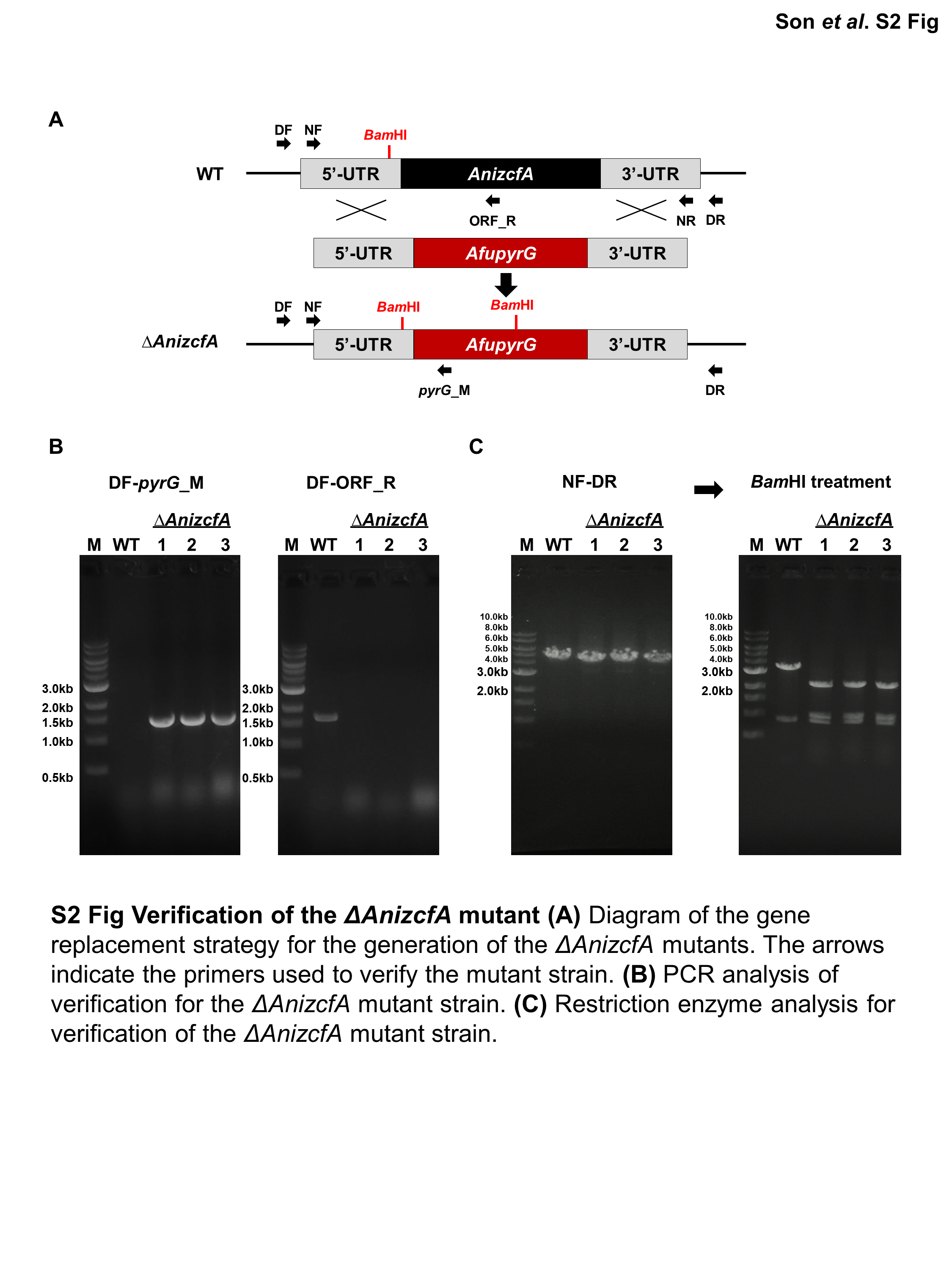

Supplement: S2 Fig — (A) Diagram of the strategy used to generate the ΔAnizcfA mutant strain. Arrows indicate the primers used to verify the mutant strain. (B) PCR verification of the ΔAnizcfA mutant strain. (C) Restriction enzyme digestion verification of the ΔAnizcfA mutant strain. (TIF) [file pone.0228643.s002.TIF]

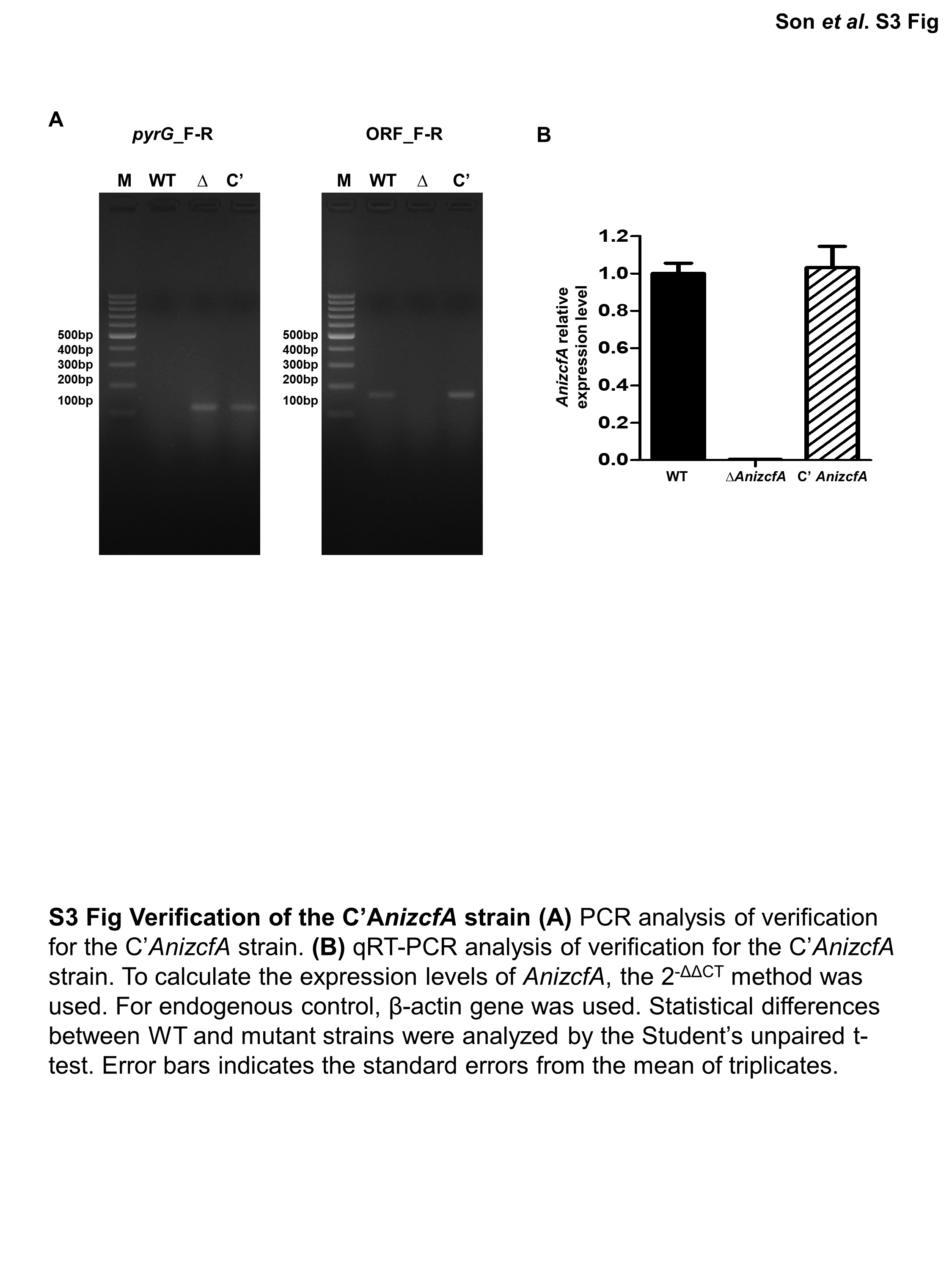

Supplement: S3 Fig — (A) PCR verification of the C’AnizcfA strain. (B) qRT-PCR verification of the C’AnizcfA strain. To calculate the expression levels of AnizcfA, the 2-ΔΔCT method was used, with β-actin as an endogenous control. Statistical differences between WT and mutant strains were analyzed by the Student’s unpaired t-test. Error bars indicate the standard error of the mean in triplicate samples. (TIF) [file pone.0228643.s003.TIF]

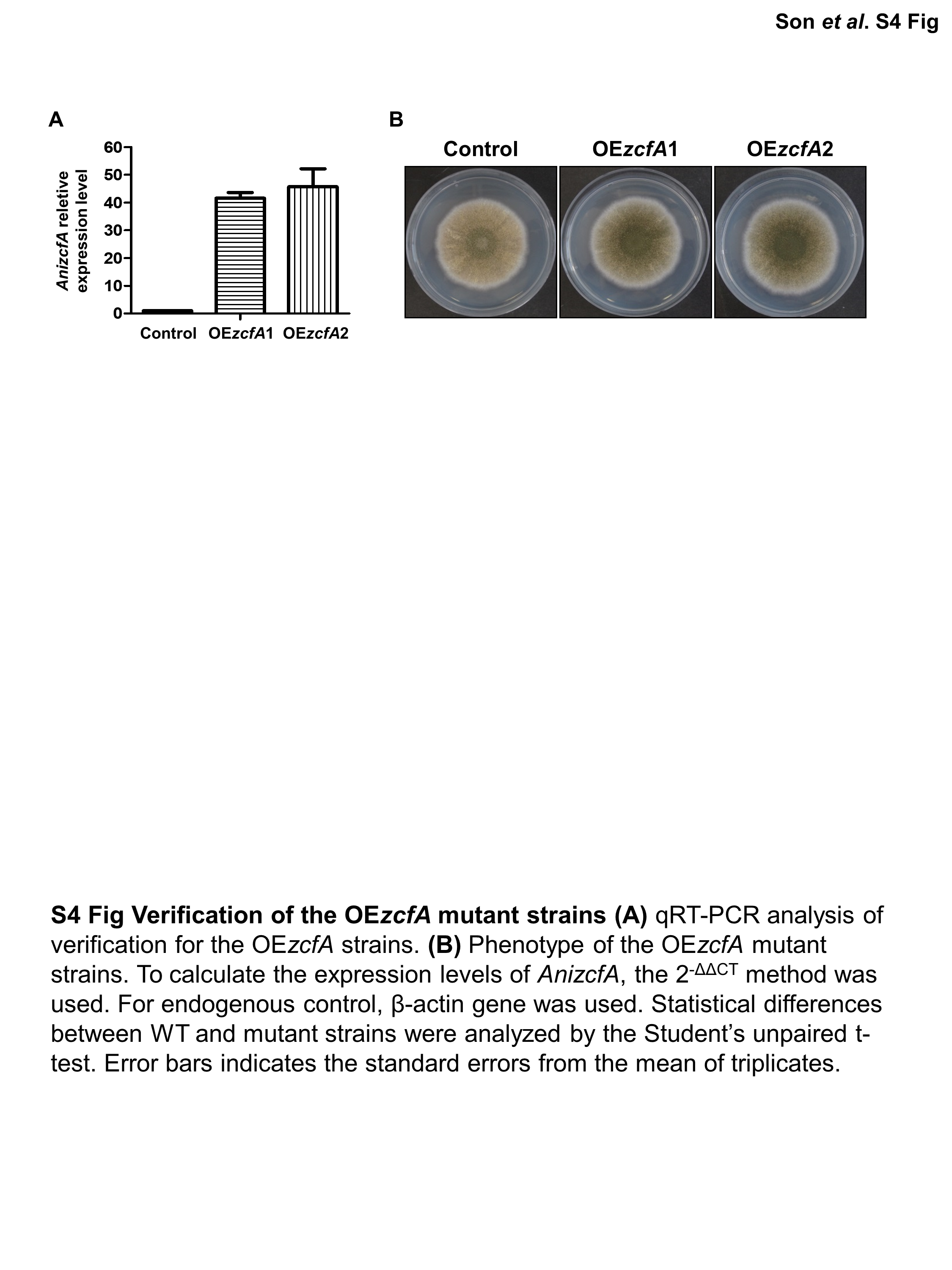

Supplement: S4 Fig — (A) qRT-PCR verification of the OEzcfA strains. (B) Phenotype of the OEzcfA mutant strains. To calculate the expression levels of AnizcfA, the 2-ΔΔCT method was used, with β-actin as an endogenous control. Statistical differences between WT and mutant strains were analyzed by the Student’s unpaired t-test. Error bars indicates the standard error of the mean in triplicate samples. (TIF) [file pone.0228643.s004.TIF]

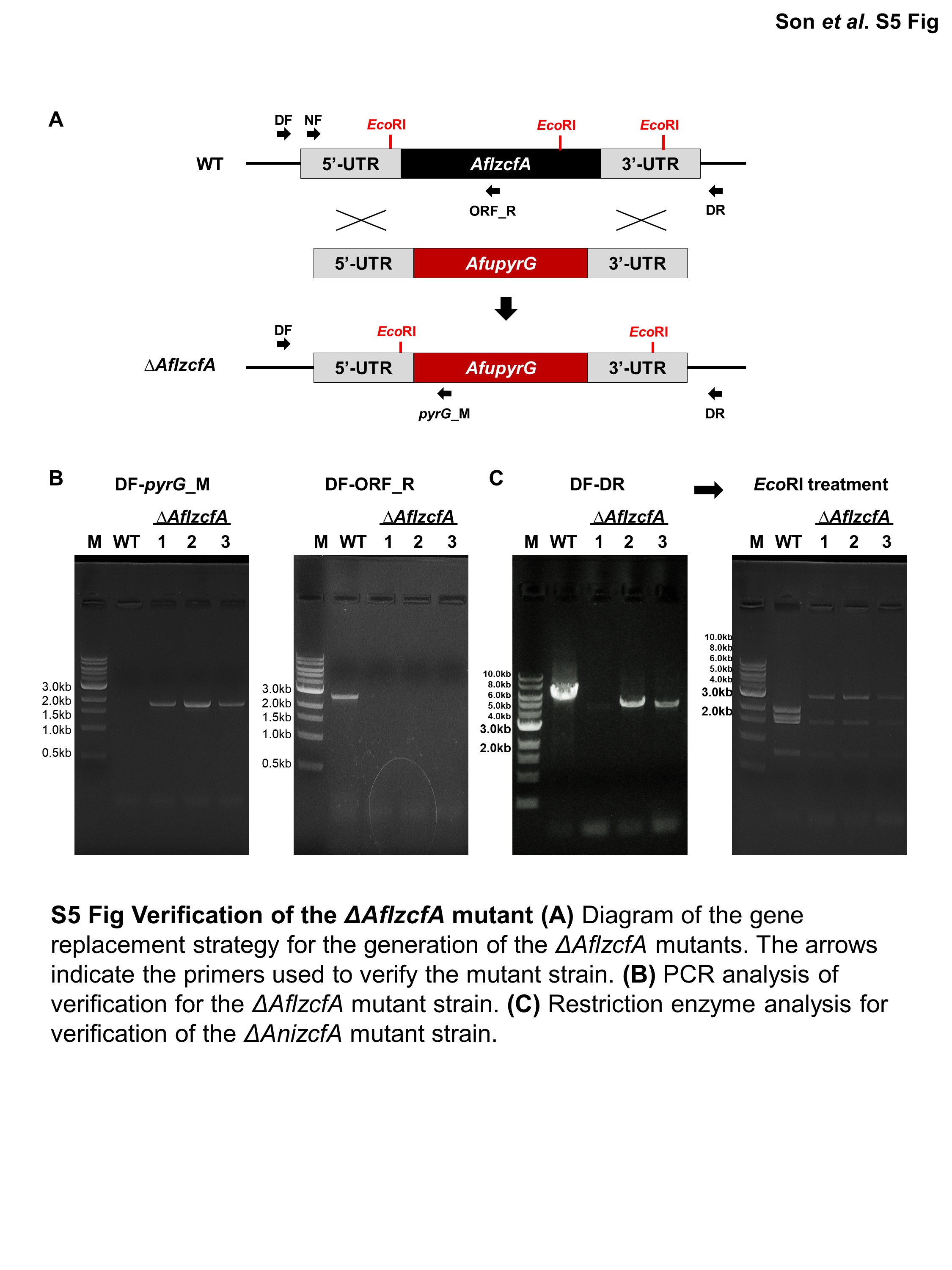

Supplement: S5 Fig — (A) Diagram of the strategy for to generate the ΔAflzcfA mutants. Arrows indicate the primers used to verify the mutant strain. (B) PCR verification of the ΔAflzcfA mutant strain. (C) Restriction enzyme digestion verification of the ΔAnizcfA mutant strain. (TIF) [file pone.0228643.s005.TIF]

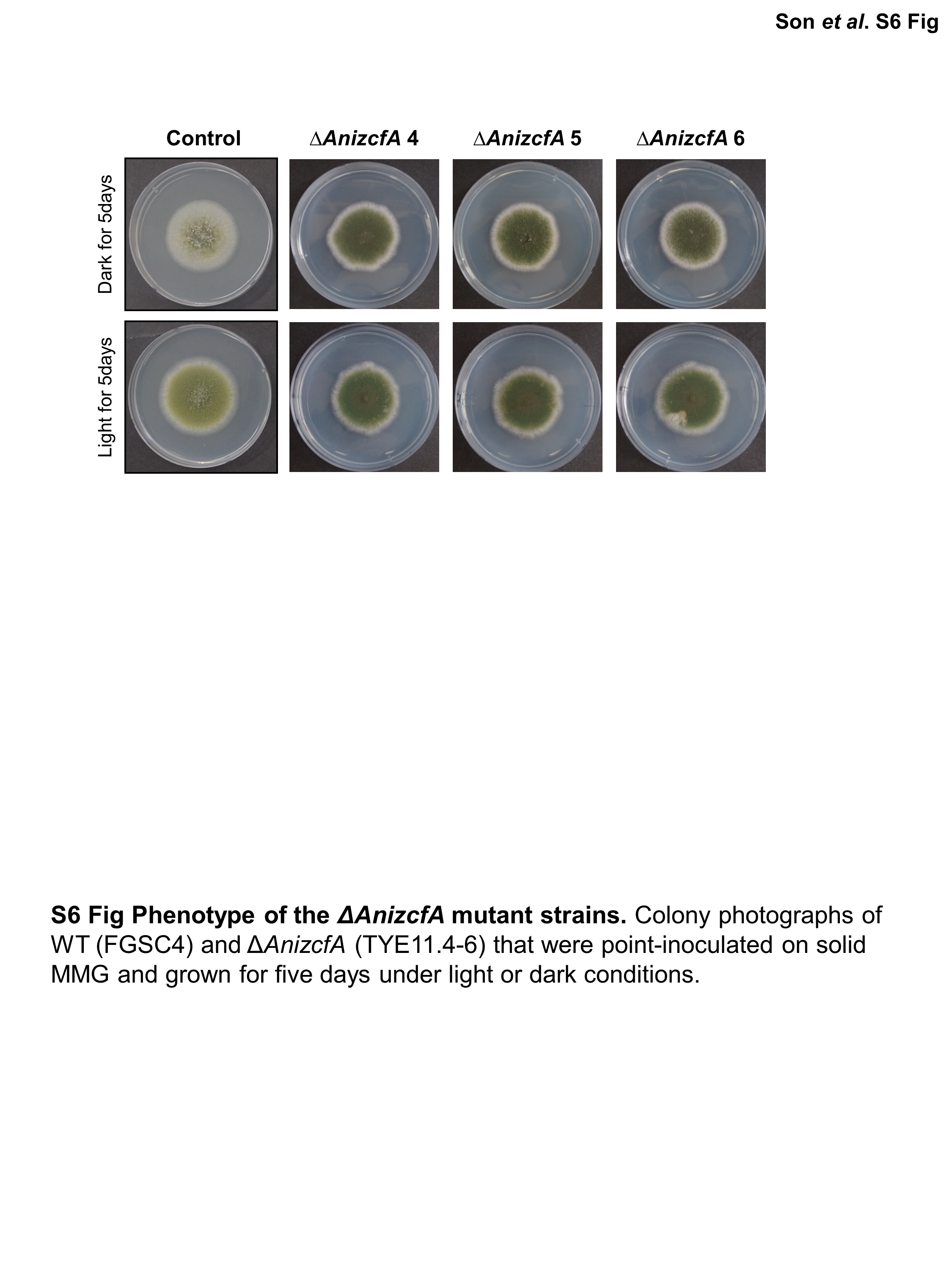

Supplement: S6 Fig — Colony photographs of WT (FGSC4) and ΔAnizcfA (TYE11.4–6) that were point-inoculated on solid MMG and grown for five days under light or dark conditions. (TIF) [file pone.0228643.s006.TIF]

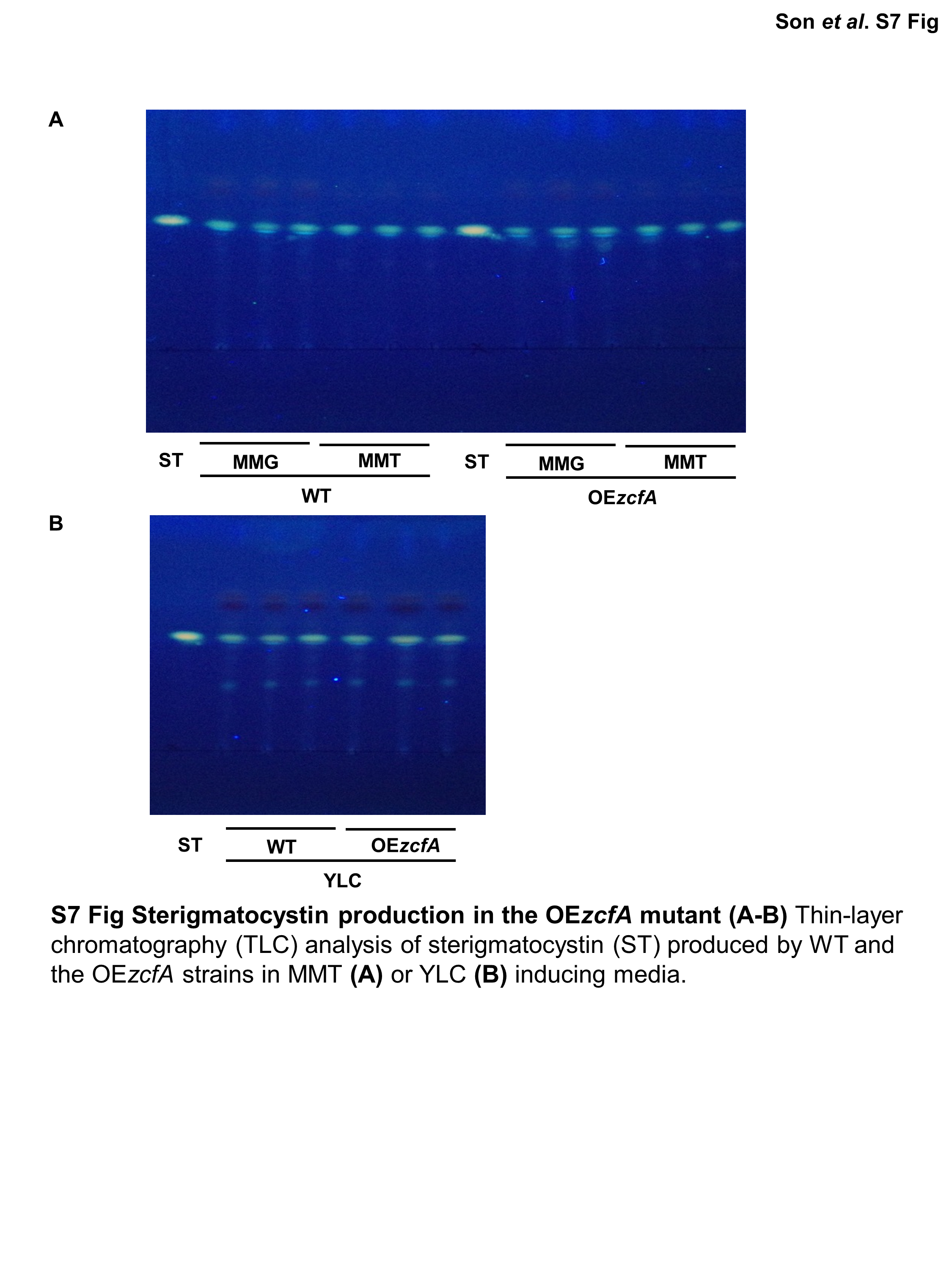

Supplement: S7 Fig — (A-B) Thin-layer chromatography (TLC) analysis of sterigmatocystin (ST) produced by WT and OEzcfA strains in MMT (A) or YLC (B) inducing media. (TIF) [file pone.0228643.s007.TIF]

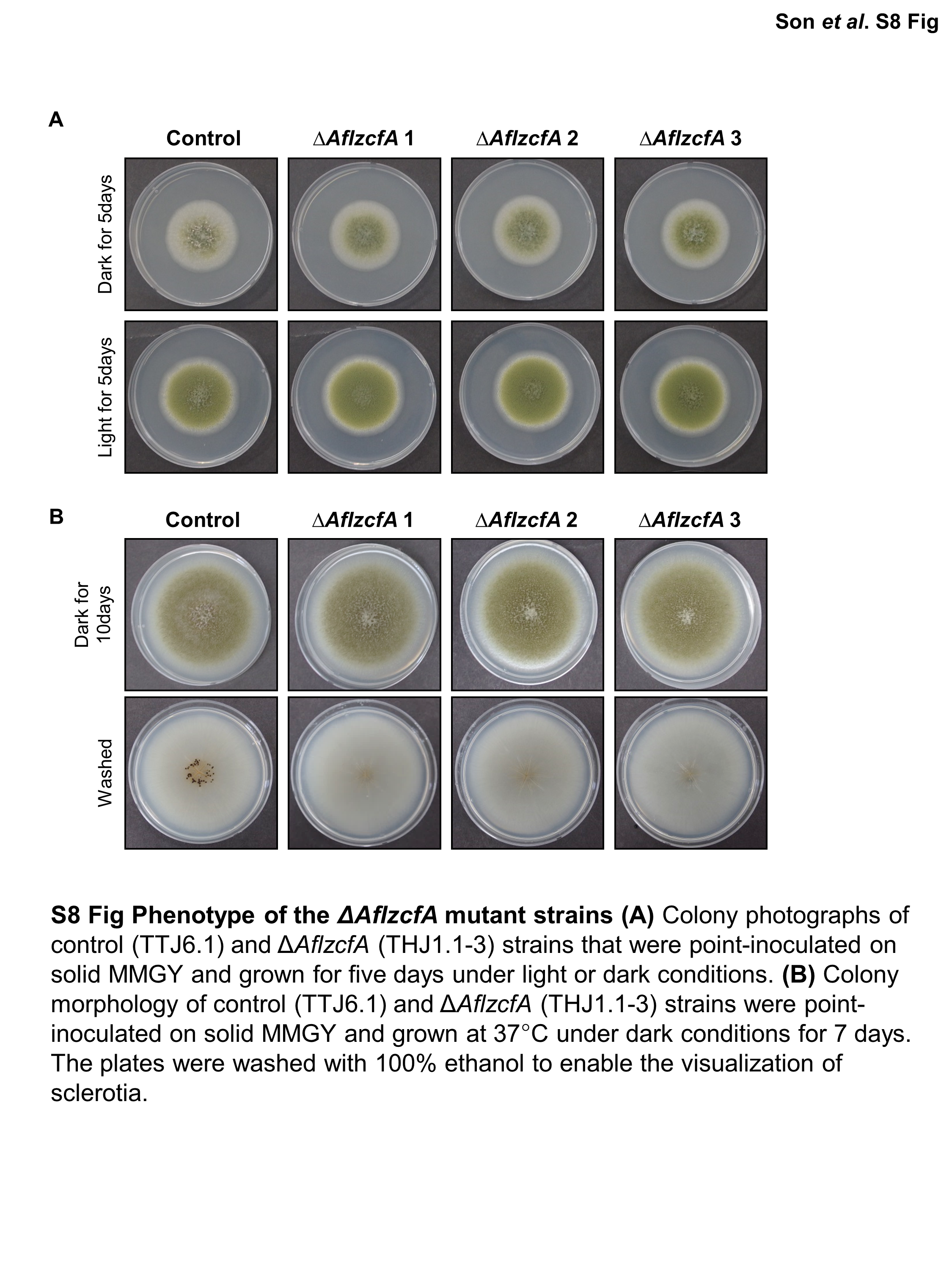

Supplement: S8 Fig — (A) Colony photographs of control (TTJ6.1) and ΔAflzcfA (THJ1.1–3) strains that were point-inoculated on solid MMGY and grown for five days under light or dark conditions. (B) Colony morphology of control (TTJ6.1) and ΔAflzcfA (THJ1.1–3) strains were point-inoculated on solid MMGY and grown at 37°C under dark conditions for 7 days. The plates were washed with 100% ethanol to visualize sclerotia. (TIF) [file pone.0228643.s008.TIF]
